# Cirrhotic Liver Sustains In Situ Regeneration of Acellular Liver Scaffolds after Transplantation into G-CSF-Treated Animals

**DOI:** 10.3390/cells12070976

**Published:** 2023-03-23

**Authors:** Marlon Lemos Dias, Inês Julia Ribas Wajsenzon, Gabriel Bastos Naves Alves, Bruno Andrade Paranhos, Cherley Borba Vieira Andrade, Victoria Regina Siqueira Monteiro, Raysa Maria Reis de Sousa, Evelyn Nunes Goulart da Silva Pereira, Karine Lino Rodrigues, Anissa Daliry, Debora Bastos Mello, Regina Coeli dos Santos Goldenberg

**Affiliations:** 1 Grupo de Estudos do Fígado, Centro de Pesquisas em Medicina de Precisão, Instituto de Biofísica Carlos Chagas Filho, Universidade Federal do Rio de Janeiro (UFRJ), Rio de Janeiro 21941-902, Brazil; 2 Instituto Nacional de Ciência e Tecnologia em Medicina Regenerativa, INCT-REGENERA, Universidade Federal do Rio de Janeiro (UFRJ), Rio de Janeiro 21941-902, Brazil; 3 Departamento de Histologia e Embriologia, Universidade do Estado do Rio de Janeiro (UFRJ), Rio de Janeiro 21949-900, Brazil; 4 Instituto de Biofísica Carlos Chagas Filho, Universidade Federal do Rio de Janeiro (UFRJ), Rio de Janeiro 21941-902, Brazil; 5 Laboratório de Investigação Cardiovascular, Instituto Oswaldo Cruz, FIOCRUZ, Rio de Janeiro 21041-250, Brazil; 6 Centro Nacional de Biologia Estrutural e Bioimagem, CENABIO, Universidade Federal do Rio de Janeiro (UFRJ), Rio de Janeiro 21941-902, Brazil

**Keywords:** decellularized extracellular matrix, decellularized liver, ductular reaction, G-CSF, in vivo recellularization, liver cirrhosis, liver fibrosis, liver regeneration, tissue engineering

## Abstract

Acellular liver scaffolds (ALS) produced by decellularization have been successfully explored for distinct regenerative purposes. To date, it is unknown whether transplanted ALSs are affected by cirrhotic livers, either becoming cirrhotic themselves or instead remaining as a robust template for healthy cell growth after transplantation into cirrhotic rats. Moreover, little is known about the clinical course of recipient cirrhotic livers after ALS transplantation. To address these questions, we transplanted ALSs into cirrhotic rats previously treated with the granulocyte colony-stimulating factor. Here, we report successful cellular engraftment within the transplanted ALSs at 7, 15, and 30 days after transplantation. Recellularization was orchestrated by liver tissue cell activation, resident hepatocytes and bile duct proliferation, and an immune response mediated by the granulocyte components. Furthermore, we showed that transplanted ALSs ensured a pro-regenerative and anti-inflammatory microenvironment, attracted vessels from the host cirrhotic tissue, and promoted progenitor cell recruitment. ALS transplantation induced cirrhotic liver regeneration and extracellular matrix remodeling. Moreover, the transplanted ALS sustained blood circulation and attenuated alterations in the ultrasonographic and biochemical parameters in cirrhotic rats. Taken together, our results confirm that transplanted ALSs are not affected by cirrhotic livers and remain a robust template for healthy cell growth and stimulated cirrhotic liver regeneration.

## 1. Introduction

Liver decellularization produces a non-immunogenic, three-dimensional biological scaffold consisting of an intricate network of structural and non-structural macromolecules from the extracellular matrix (ECM) [1]. The liver ECM plays an important role in tissue maintenance, homeostasis, and regeneration [2]. In addition, the ECM orchestrates key cellular behaviors that impact cell support, shape, movement, adhesion, communication, function, and cellular response [1,2].

Accordingly, acellular liver scaffolds (ALS) produced by decellularization have been successfully applied to distinct regenerative aims, and beneficial results were achieved when these scaffolds were transplanted in vivo [3,4,5]. While several studies have focused on the recellularization steps before transplantation, ALS transplantation itself has only been partially explored. Although total orthotopic transplantation of ALS would not support the metabolic demands of patients after transplantation, other alternatives can be explored to find new therapeutic tools in the fields of hepatology and regenerative medicine [6]. For example, ALS could be applied after partial hepatectomy to increase the limit of liver resection [6,7] or the scaffold could be used in partial orthotopic transplantation as a way to create a bioartificial liver that ensures metabolic functions while the patient’s own liver regenerates [6,8].

The first report of ALS orthotopic transplantation was by Zhang and colleagues in 2015, which provided a proof of concept that decellularized matrices can be transplanted into recipient mice [9]. Subsequently, Naeem and colleagues performed ALS transplantation after lobectomy and confirmed in situ recellularization when the ALS was transplanted into recipient rats [10]. Shimoda and colleagues evaluated the potential contribution of ALS transplantation to hepatic regeneration after hepatectomy in recipient pigs [7]. Taken together, these studies showed that ALS could be recellularized by healthier cells and promoted liver regeneration after hepatectomy when transplanted into mice [9], rats [10], and pigs [7]. Despite these significant advances, the ALS recellularization process and the body’s ability to repopulate the scaffold was investigated only in healthy recipient animals. Therefore, as a next step, it is necessary to investigate cellular recruitment, cell engraftment, post-transplantation angiogenesis and potential therapeutic improvement after ALS transplantation in liver disease models. 

To achieve this, we combined in vivo and ex vivo techniques, tissue engineering tools, and a therapeutic approach that involved the granulocyte colony stimulating factor (G-CSF), which is known to stimulate bone marrow cell mobilization. To our knowledge, this study is the first to explore ALS transplantation in G-CSF-pretreated cirrhotic animals. Our strategy is attractive because G-CSF has already been employed in clinical practice for bone marrow cell mobilization, and cumulative evidence has shown that G-CSF exerts beneficial effects in liver disease [11,12,13]. Therefore, the purpose of this study was to investigate whether ALS could be recellularized by healthy cells when transplanted into cirrhotic rats pre-treated with G-CSF, and to elucidate the regenerative events. Accordingly, we also aimed to investigate whether ALS promotes the formation of functional liver tissue in situ and improves the status of the cirrhotic liver after transplantation. In this work, we investigated the hypothesis that ALS could be transplanted directly into a diseased recipient animal, relying entirely on the scaffold’s ability to promote recruitment, adhesion, and repopulation of resident cells to regenerate endogenous liver tissue. We observed that, even under liver disease conditions, the body can act to promote ALS recellularization.

## 2. Material and Methods

### 2.1. Experimental Design

**Figure 1:**
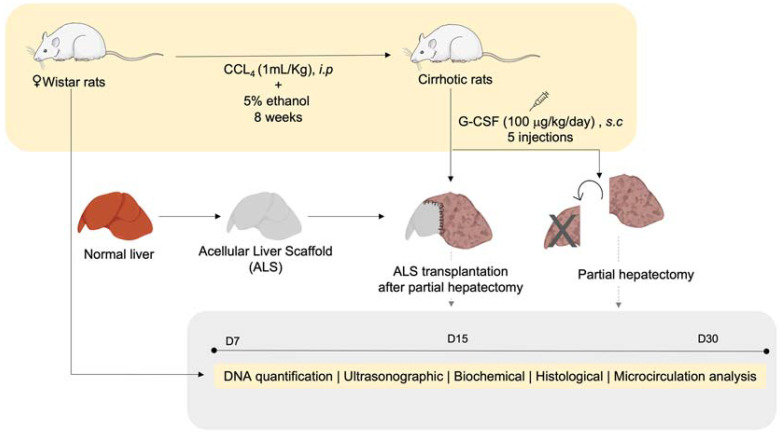
**Experimental design.** The animals were subjected to liver cirrhosis induction by intraperitoneal injections of carbon tetrachloride (CCl_4_) in association with 5% ethanol in drinking water. At the same time, the donor animals underwent surgical excision of the liver. The livers were decellularized to generate an acellular liver scaffold (ALS). Five consecutive days before transplantation, the recipient animals (cirrhotic animals) received subcutaneous injections of granulocyte colony-stimulating factor (G-CSF). The ALS was then transplanted, and ultrasonographic, microcirculation, biochemical, and histological analyses were performed 7, 15, and 30 days after transplantation.

Female Wistar rats were subjected to liver cirrhosis induction by intraperitoneal injections of carbon tetrachloride (CCl_4_) in association with 5% ethanol in drinking water. At the same time, the donor animals underwent surgical excision of the liver. Each donor liver was decellularized to generate an ALS. Five consecutive days before transplantation, the recipient animals (cirrhotic animals) received subcutaneous injections of granulocyte colony-stimulating factor (G-CSF). Each ALS was then transplanted into a cirrhotic recipient, and ultrasonographic, microcirculation, biochemical, and histological analyses were performed 7, 15, and 30 days after transplantation. The experimental design is illustrated in Figure 1.

### 2.2. Animals

This study was approved (097/20) and followed the animal care guidelines of the Animal Ethics Committee of the Health Science Center of the Federal University of Rio de Janeiro, Brazil. Forty-one female Wistar rats, ranging in age from 8 to 14 weeks, were used. The animals were randomly distributed into the following four groups: liver donor rats (*n* = 9), control rats (*n* = 3), cirrhotic transplanted rats (Tx; *n* = 20), and cirrhotic and partial hepatectomized rats (PH; *n* = 9). Animals were kept on a 12 h light/dark cycle, 25 °C ambient temperature, and 55 ± 5% humidity. During the experiment, the rats were fed standard pellets and water *ad libitum* and their body weight was monitored weekly. Anesthesia was provided by inhalation of 3–4% isoflurane (Isoforine^®^, Cristália, São Paulo, Brazil) and maintained by inhalation of 1–2% isoflurane. All rats were given oxygen at a dose of 0.3–0.5 L/min.

### 2.3. Liver Cirrhosis Induction

Female Wistar rats received CCl_4_ 1 mL/kg diluted in olive oil (1:1) intraperitoneally, three times a week every other day over 8 weeks. In addition, the animals had access to drinking water containing 5% ethanol (*v*/*v*) “Caninha da roça” (Indústria de Bebidas Paris LTDA, Rio de Janeiro, Brazil) ad libitum. The control group received 1 mL/kg of olive oil intraperitoneally, three times a week, for 8 weeks.

### 2.4. Granulocyte Colony-Stimulating Factor Treatment

Cirrhotic rats received G-CSF (100 μg/kg/day) (Filgrastine, Blau Farmacêutica, Belo Horizonte, Brazil) subcutaneously for five consecutive days before partial hepatectomy or ALS transplantation to stimulate bone marrow cell mobilization. Blood samples (500 µL) were collected in EDTA K2 microtubes (Vacuplast, São Paulo, Brazil) and subjected to hemogram analysis in a hematology analyzer on days 0, 3, and 5 after G-CSF treatment. At the same time points, hematology sections were stained with Panoptic Fast Staining (Laborclin, Paraná, Brazil) using 10 μL of blood. The images were obtained by using a Pannoramic MIDI II microscope and scanner (3DHISTECH Ltd., Budapest, Hungary).

### 2.5. Biochemical Analysis

Blood samples (500 μL) were collected by cardiac puncture in a clot activator gel microtube (Vacuplast, São Paulo, Brazil) before and after cirrhosis induction. In addition, blood samples were also collected before and after 7, 15 and 30 days post-transplantation. Then, the blood samples were centrifugated at 1400 g for 10 min and the serum was stored at −20 °C. Serum levels of albumin (ALB), alanine aminotransferase (ALT), lactate dehydrogenase (LDH), alkaline phosphatase (AP), urea, total bilirubin (TB), and aspartate aminotransferase (AST) were determined with a semi-automatic biochemical analyzer Bio 200 (Bioplus, Rio de Janeiro, Brazil), using an ALB detection kit (Ref. 19, Labtest, Minas Gerais, Brazil), ALT kit (Ref. 108, Labtest, Minas Gerais, Brazil), LDH kit (Ref. 138-1/50, Labtest, Minas Gerais, Brazil), AP kit (Ref. 79-4, Labtest, Minas Gerais, Brazil), Urea kit (Ref. 104-4/50, Labtest, Minas Gerais, Brazil), TB kit (Ref. 94-1, Labtest, Minas Gerais, Brazil) and AST detection kit (Ref. 109, Labtest, Minas Gerais, Brazil).

### 2.6. Liver Procurement

The animals (*n* = 9) were heparinized with 100 UI of heparin (Hemofol^®^, Cristália, São Paulo, Brazil) 15 min before the surgical procedures. A transverse abdominal incision followed by laparotomy was performed. Then, the portal vein (PV) was isolated and cannulated with the aid of a 24-gauge catheter (Angiocatch^®^, BD, São Paulo, Brazil). After PV cannulation, 5 mL of Custodiol (Contatti Medical, Porto Alegre, Brazil) solution was perfused into the liver. Subsequently, the liver was carefully excised and placed in a 60 mm sterile Petri dish.

### 2.7. Liver Decellularization

Harvested rat livers (*n* = 9) were decellularized as previously described [14] using a peristaltic pump (Masterflex Cole Parmer L/S, Model 7522-20) at a speed of 3 mL/min. Briefly, the liver was subjected to continuous perfusion with water (2 h) and 1% (*v*/*v*) Triton X-100 solution (Sigma-Aldrich, Saint Louis, MO, USA) (2 h) through the PV. Subsequently, a 1% sodium dodecyl sulfate (SDS) (Synth, São Paulo, Brazil) solution was perfused for 18–24 h. After washing with distilled water (overnight), the ALS was perfused with a solution containing 1% amphotericin b, 1% penicillin, and streptomycin (Sigma-Aldrich, Saint Louis, MO, USA) for 1 h.

### 2.8. Vascular Tree Evaluation

Macroscopic analysis of the ALS vasculature was performed by perfusion of toluidine blue and phenol red. Initially, the portal vein and bile duct were cannulated with the aid of a 24G catheter (Angiocatch^®^, BD, São Paulo, Brazil). Then, 1 mL of toluidine blue was infused through the portal vein and 1 mL of phenol red through the bile duct. The microscopic analysis was performed by using an Olympus BX150WI microscope (Center Valley, PA, USA).

### 2.9. Histological Analysis

Biopsies from normal livers and ALSs were fixed in formalin (4%) for 48 h. Then, the biopsies were paraffin-embedded and sectioned (5 μm) for the following analyses (*n* = 3 each): to investigate the morphology, the sections were stained with hematoxylin and eosin (H&E) (Merck, São Paulo, Brazil); to investigate the deposition of the ECM, tissue sections were stained with Gomori’s trichrome, Sirius red, and reticulin. In addition, to investigate glycogen storage, tissue sections were stained with periodic acid–Schiff (PAS) (Merck, São Paulo, Brazil). The images were obtained by using a Pannoramic MIDI II microscope and scanner (3DHISTECH Ltd., Hungary) or an Olympus BX53 polarizing microscope (Olympus Corporation, Japan).

### 2.10. Immunohistochemical and TUNEL Analyses

For the immunohistochemical analyses, following deparaffinization and rehydration, the sections were exposed to hydrogen peroxide (3%) diluted in phosphate-buffered saline (PBS) (30 min). Excess peroxide was removed with PBS + Tween 0.2% (2 min). Antigen retrieval was achieved by immersing the slides in sodium citrate buffer (pH 6.0) in a steam cooking appliance at 96 °C (Fun Kitchen, São Paulo, Brazil) (30 min). Then, the slices were immersed in ice for 30 min, followed by two wash steps with PBS + Tween 0.2% (2 min each). The tissue sections were incubated in bovine serum albumin (3%) in PBS for 1 h to block non-specific antibody binding. The slides were then incubated with primary antibodies for Ki67 (1:100; M3064; Spring Bioscience, Pleasanton, CA, USA) and anti-Cytokeratin 7 (CK7) (1:100; M3524, Spring Bioscience, Pleasanton, CA, USA) overnight at 4 °C. Tissue sections with the absence of primary antibodies were used as a control. The next day, the slides were washed with PBS + Tween 0.2% (3 min) and then incubated with a biotin-conjugated secondary antibody (Vectastain universal quick HRP Kit, Vector, Newark, CA, USA) for 10 min. After incubation with streptavidin (Vectastain universal quick HRP Kit, Vector, USA) for 5 min, the reaction was revealed with 3,3-diaminobenzidine (DAB) (SPD-060- Spring Bioscience, Pleasanton, CA, USA) for 2 min and stopped with water. Finally, the sections were stained with hematoxylin for 1 min. The images were obtained by using a Pannoramic MIDI II microscope and scanner (3DHISTECH Ltd., Hungary) or an Olympus BX53 microscope (Olympus Corporation, Japan). TUNEL analysis was used for apoptotic nuclei detection, using the ApopTag^®^ In Situ Peroxidase Detection Kit (Merck Millipore, Saint Louis, MO, USA), according to the manufacturer’s recommendations. For quantification of Ki67, the area of each region (graft/ALS and cirrhotic liver) of immunostained tissue sections was measured using the free-drawing tool of Image J software (National Institutes of Health, Bethesda, MD, USA). Five representative images (100× objective) derived from three experimental tissue sections (*n* = 3 each) were used for quantification analysis. The positive nuclei were quantified using CountThings software. The quantifications were conducted by a blinded examiner.

### 2.11. Immunofluorescence Analysis

For immunofluorescence analysis, paraffin-embedded tissue sections were subjected to deparaffinization and rehydration steps. Then, antigen retrieval was achieved by immersing the slides in sodium citrate buffer (pH 6.0) in a steam cooking appliance at 96 °C (Fun Kitchen, São Paulo, Brazil) (30 min). Then, the slides were immersed in ice for 30 min, followed by two wash steps with PBS + Tween 0.2% (2 min each). Autofluorescence block was achieved by ammonium chloride 0.5 mM. After that, the tissue sections were washed with PBS three times. The tissue sections were incubated in bovine serum albumin (3%) in PBS for 1 h to block non-specific antibody binding. Tissue slices were then incubated with primary antibodies for anti-Cytokeratin 19 (CK19) (1:100; Ab15463, Abcam, Boston, MA, USA), anti F4/80 (1:100; Ab6640, Abcam, Boston, MA, USA) and anti CD11b (1:100; ab8878, Abcam, Boston, MA, USA) overnight, followed by washing and secondary incubation in secondary antibodies diluted in PBS/BSA 3% (Alexa Fluor 488 anti-rat, 1:500; A11006, Life technologies; Alexa Fluor 488 anti-mouse, 1:500, A11017, Invitrogen; Alexa Fluor 488 anti-sheep, 1:500, A11015, Invitrogen and Alexa Fluor 594 anti-rabbit, 1:500, A11012, Molecular Probes). The tissue sections were washed and counterstained with 4′,6-diamidino-2-phenylindole (DAPI; 1:500). Images were acquired by using a Zeiss Apotome confocal microscope (Zeiss, Germany).

### 2.12. Scanning and Transmission Electron Microscopy (SEM and TEM) Analysis

Normal livers or the ALS sections were subjected to washing steps (3x) with 0.2 M PBS (pH 7.2) (LGC Biotecnologia, Rio de Janeiro, Brazil) and fixed in 0.2 M sodium cacodylate buffer (Electron Microscopy Science, USA) containing 2.5% glutaraldehyde (Sigma-Aldrich, USA) at 4 °C for 24 h prior to electron microscopic observation. To remove the glutaraldehyde, the specimens were washed (3 times) in sodium cacodylate buffer (pH 7.2), and then they were dehydrated through an ascending alcohol series (30%, 50%, 70%, 90%, and 100%). Subsequently, the specimens were dried in a critical point dryer (CPD2; Tousimis, Cambridge, MA, USA) and coated with gold in an ion-sputtering apparatus (Sputter Coater 108; Cressington, Watford, UK). The fragments were observed using a scanning electron microscope (1450 VP, LEO Electron Microscopy Ltd., Clifton Road, UK). In addition, semi-thin tissue sections (1 μm) were prepared and stained with toluidine blue and then images were obtained by using an Olympus BX53 microscope (Olympus Corporation, Japan). The acquired images were qualitatively analyzed. For TEM analysis, the recipient cirrhotic liver and graft tissues were processed and then quantitatively analyzed using a JEM1011 (Jeol, Akishima, Tokyo, Japan) microscope. The cirrhotic liver and graft specimens were washed in PBS and fixed for 1 h in a solution containing 2.5% glutaraldehyde in 0.1 M sodium cacodylate buffer (pH 7.2) plus 3.5% sucrose. Then, the samples were washed for 10 min in the same buffer. This washing step was repeated 3 times. The tissue was fixed for 1 h in a 1% osmium tetroxide (OsO4) solution in 0.1 M sodium cacodylate buffer (pH 7.2) plus 3.5% sucrose, dehydrated in an acetone series (30%, 50%, 70%, 90% and 100%) and embedded in Poly/Bed(r) 812 resin (Ted Pella Inc., Redding, CA, USA). After polymerization, ultrathin sections were obtained and contrasted with uranyl acetate–lead citrate for ultrastructural observation.

### 2.13. DNA Content Analysis

DNA was isolated from 25 mg (dry tissue) of control liver, acellular liver scaffold and transplanted liver tissue (*n* = 3) and detected by DNeasy® Blood & Tissue Kit (Qiagen, Hilden, Germany). Then, the samples were read at Nanophotometer Pearl^®^ (IMPLEN, München, Germany). 

### 2.14. ALS Orthotopic Transplantation

The ALS was first perfused with Custodiol (5 mL) and 50 μg/mL hepatocyte growth factor (HGF, Sigma-Aldrich, USA) and then orthotopically transplanted into a cirrhotic recipient Wistar rat, as previously described [12]. The recipient rat previously received granulocyte colony-stimulating factor G-CSF (100 μg/kg/day) (Filgrastine, Blau Farmacêutica, Belo Horizonte, Brazil) subcutaneously for five consecutive days before ALS transplantation to stimulate bone marrow cell mobilization. Blood samples (500 µL) were collected in EDTA K2 microtubes (Vacuplast, Brazil) and subjected to hemogram analysis in a hematology analyzer on days 0, 3, and 5 after G-CSF treatment. At the same time points, hematology sections were stained with Panoptic Fast Staining (Laborclin, Paraná, Brazil) using 10 μL of blood. The images were obtained by using a Pannoramic MIDI II microscope and scanner (3DHISTECH Ltd., Hungary). To perform orthotopic transplantation, recipient animals were subjected to partial median lobe hepatectomy (10%) and subsequently ALS transplantation, after continuous suture with 6–0 silk suture. After transplantation, the animal received 100 μL of tramadol for three days. Only partial hepatectomized rats (*n* = 9) were used as a control group. The hepatectomized and transplanted animals were euthanized 7, 15 and 30 days after transplantation. After euthanasia, blood samples were collected, the graft length was measured by a pachymeter, and then tissue sections derived from the cirrhotic liver and the graft were submitted for histological analysis.

### 2.15. Ultrasound Analysis

The animals were anesthetized, subjected to abdominal hair shaving, and then examined in the supine position using a Vevo 2100 with a transducer with the frequency 13–24 MHz (MS250; Visual Sonics, Toronto, ON, Canada). The liver and kidneys were evaluated using transverse and longitudinal scans. The evaluated parameters were the portal vein diameter and blood flow velocity. To assess the organ status, liver aspects, echogenicity and the echographic relationship between the liver and renal cortex were analyzed. Ultrasound analyses were performed before and 8 weeks after cirrhosis induction and at 7, 15, and 30 days after transplantation or partial hepatectomy.

### 2.16. Microcirculation Analysis

For intravital microscopy of the transplanted ALS, normal and cirrhotic Wistar rats were anesthetized by intraperitoneal (i.p.) injection of ketamine (100 mg/kg) and xylazine (10 mg/kg). To exteriorize the liver, midline and left subcostal incisions were performed. The hepatic ligaments were dissected, and the median liver lobe was then exteriorized, placed on a glass disk, and covered with a glass slide for microcirculation analysis. Using a 10× ocular and 10× objective (Olympus BX150WI; Center Valley, PA, USA), images were displayed on a television monitor and recorded by a digital video recorder (DP73; Olympus, MA, USA) for off-line analysis with the Cellsens standard 1.9 software program (Olympus, MA, USA). Hepatic microvascular blood flow was measured using a Laser Speckle Contrast Imaging apparatus (Pericam PSI system, Perimed, Sweden), which provides a microcirculatory perfusion index proportional to the concentration and mean velocity of red blood cells used to assess microvascular blood flow in real time. To perform this analysis, the animals were held on a stable surface and placed under a laser light system with image contrast at a wavelength of 785 nm for continuous measurement of tissue blood perfusion in real time. The distance between the scan head and the cirrhotic liver and ALS surface was approximately 10 cm. Relative liver blood flow in all animal groups was expressed in arbitrary perfusion units (APUs). In cirrhotic livers and grafts, the number of vitamin A-positive hepatic stellate cells (HSCs) was determined as the number of fluorescent cells derived from vitamin A autofluorescence.

### 2.17. Statistical Analysis

GraphPad Prism 9 (GraphPad Software, La Jolla, CA, USA) was used to conduct statistical analyses. Descriptive data are presented as means ± standard deviations (SD). Comparisons between the groups were performed using a paired or unpaired Student’s *t*-test, or one-way ANOVA followed by Tukey’s test or two-way ANOVA followed by Šídák’s multiple comparisons test. A value of *p* < 0.05 was used as a significance threshold.

## 3. Results

### 3.1. CCl_4_ + Ethanol Induces Liver Cirrhosis in Rats

**Figure 2:**
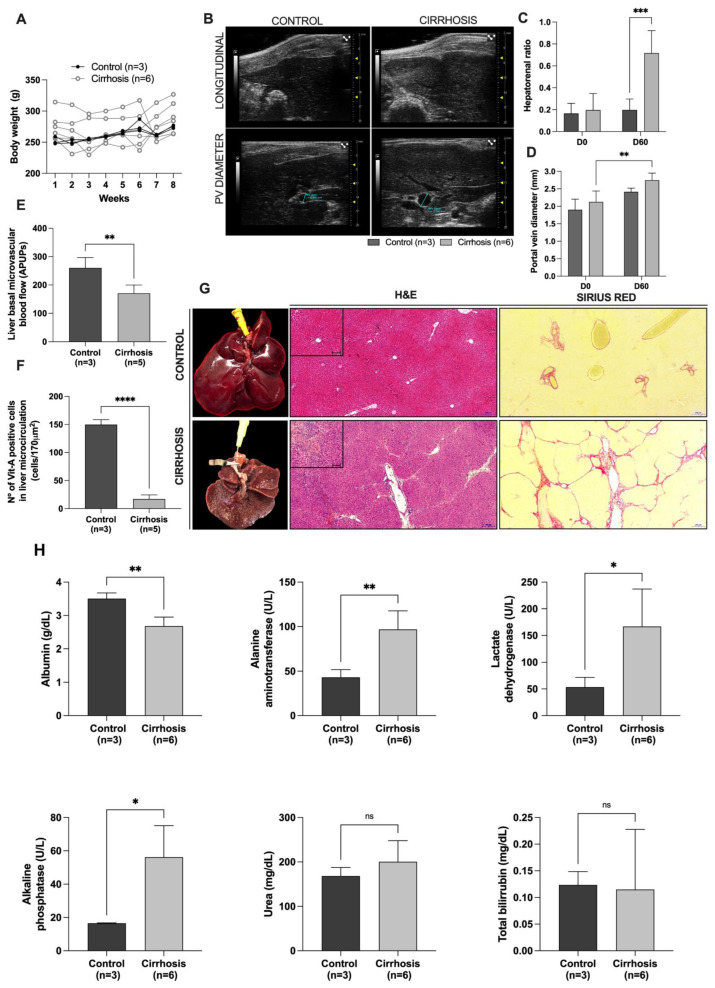
**CCl_4_ + ethanol induces liver cirrhosis in rats.** Body weight of control and cirrhotic animals (**A**). Hepatorenal ultrasound findings (**B**). Ultrasonographic images revealed similarities between liver and kidney echogenicity and an increased portal vein (PV) diameter in the cirrhosis group, whereas the liver was hyperechogenic in relation to renal parenchyma in the control group. Hepatorenal ratio before and after 60d of cirrhosis induction (**C**). A significant increase in the hepatorenal ratio was observed in the cirrhotic group 60d after CCl_4_ and ethanol administration. The PV diameter also increased in the cirrhosis group 60d after CCl_4_ and ethanol administration (**D**). The cirrhosis group also showed a significant reduction in liver basal microvascular blood flow (**E**) and in the number of vitamin A-positive cells in the liver microcirculation (**F**). Macroscopic and microscopic views of normal and cirrhotic livers (**G**). Tissue sections stained with H&E and Sirius red showed inflammatory infiltrate presence, extracellular matrix deposition and regeneration nodule scar formation in the cirrhotic liver. Serum biochemical levels of the control and cirrhotic groups (**H**). Data are presented as mean ± SD and *p* < 0.05 was considered as significant (* *p* < 0.05, ** *p* < 0.005, *** *p* < 0.001, **** *p* < 0.0001, non-significant (ns)). Two-way ANOVA followed by Šídák’s multiple comparisons test was performed in (**C**,**D**). Student’s *t*-test was performed in (**E**,**F**,**H**). Scale bars: 200 μm; 100 μm (insert images).

The first set of experiments was performed to obtain cirrhotic rats. Wistar rats subjected to 1 mL/kg CCl_4_ injections in combination with ethanol in drinking water ad libitum for 8 weeks did not show differences in body weight when compared to the control group (273.0 ± 6.36 g vs. 261.7 ± 9.92 g) (Figure 2A). In addition to body weight monitoring, we performed a non-invasive ultrasound analysis to evaluate the development of liver cirrhosis. The ultrasound analysis showed increased liver echogenicity, characterized by an extensively coarsened and heterogeneous parenchyma with an irregular hepatic surface after 8 weeks of injury induction (Figure 2B). Furthermore, a significant increase in the hepatorenal ratio was observed in cirrhotic rats 60d post-CCl_4_ plus ethanol treatment when compared to the control group (Figure 2C). The cirrhosis-induction protocol also promoted an increase in PV caliber 60d post-intoxication with CCl_4_ and ethanol (Figure 2D). The laser speckle contrast imaging analysis showed that cirrhotic livers had a significant decrease in liver microvascular blood flow compared to the control livers (Figure 2E). In addition, the number of vitamin A-positive cells in the cirrhotic livers was lower than that observed in the control livers (Figure 2F).

Macroscopic liver analysis of the cirrhotic group revealed a rigid consistency, characterized by collapsed median and left lateral lobes, with increased volume and irregular edges. A brownish parenchyma, composed of an irregular surface with regeneration nodules, was also observed (Figure 2G). Histological analysis of cirrhotic livers stained with H&E revealed a global loss of normal hepatic parenchyma architecture, marked by fibrous septa and misalignment of hepatocytes close to the central veins. Inflammatory infiltrates were also observed (insert image). Sirius red staining revealed intense collagen deposition and various fibrous septa around the portal tracts that extended into the hepatic lobule. In contrast, normal, smooth, regular, consistent, organized, and brownish-red liver parenchyma was observed in control rats (Figure 2G).

Biochemical analysis showed that cirrhotic rats had a significant decrease in albumin levels and significantly elevated serum ALT, LDH, and ALP, similar to the biochemical alterations described in human liver cirrhosis (Figure 2H). No differences were detected in the serum levels of urea or total bilirubin.

### 3.2. G-CSF Treatment Increases White Blood Cell Mobilization to Peripheral Blood in Cirrhotic Rats

**Figure 3:**
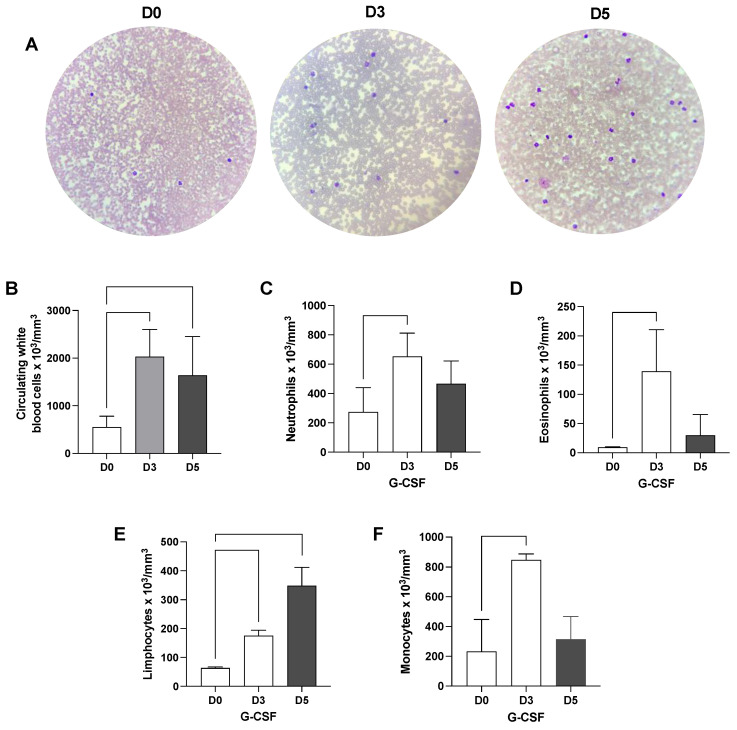
**G-CSF induces white blood cell mobilization to the peripheral blood.** Blood samples were subjected to Panoptic fast staining to reveal white blood cells before and 3 and 5 days after G-CSF treatment (**A**). Hemogram analysis (**B**–**F**) of cirrhotic rats before and after 3 and 5 injections of G-CSF. G-CSF treatment significantly enhanced leukocyte numbers in the peripheral blood of cirrhotic rats. Statistical significance was assessed by using paired Student’s *t*-test analysis (*n* = 3).

G-CSF treatment significantly increased the presence of circulating white blood cells in the peripheral blood of cirrhotic rats (Figure 3). A significant increase in cell mobilization was achieved after the third G-CSF injection and maintained after the fifth G-CSF injection (Figure 3A,B). Hemogram analysis revealed that the G-CSF treatment promoted alterations in white blood cell counts in the peripheral blood of cirrhotic rats (Figure 3C–F). Significant increases in neutrophils, eosinophils, lymphocytes, and monocytes were observed after G-CSF treatment.

### 3.3. Generation and Characterization of Acellular Liver Scaffold

**Figure 4:**
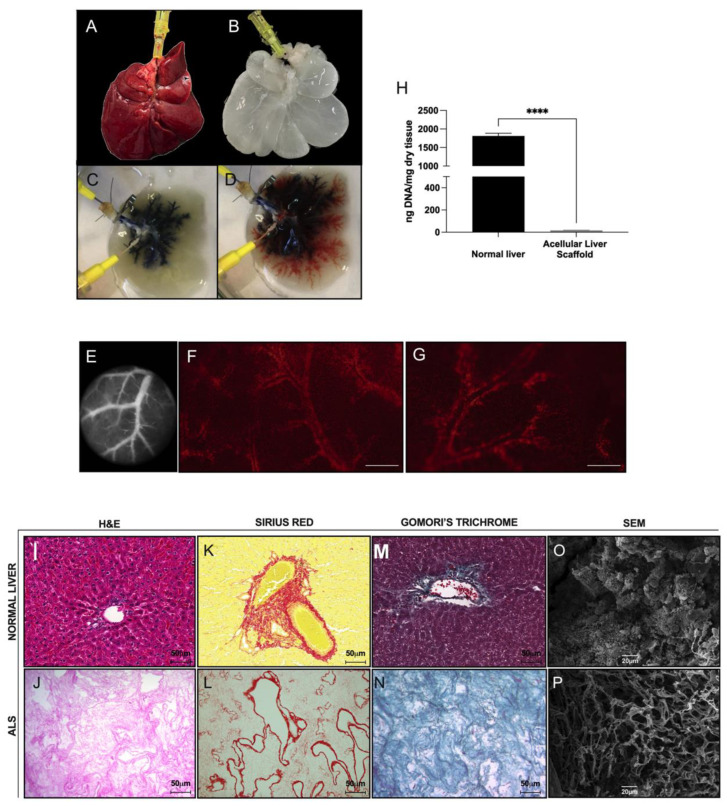
**Rat liver decellularization and acellular liver scaffold characterization**. Normal (**A**) and ALS (**B**) obtained by the decellularization process. Appearance of the vascular tree after toluidine blue and phenol red dye perfusion through the portal vein (**C**) and bile duct (**D**), respectively. A large branch of a preserved vascular tree was visualized using light (**E**) and intravital microscopy (**F**,**G**). Quantification of double-stranded DNA in ALS revealed a significant reduction in DNA compared to normal livers (*n* = 3; significant differences were assessed by applying Student’s *t*-test, **** *p* < 0.0001) (**H**). H&E staining showed pink eosinophilic staining and no remaining nuclei or cytoplasm (**J**) in the scaffold compared with the normal liver (**I**). Sirius red (**L**) and Gomori’s (**N**) staining showed red- and green-based staining, indicating the presence of ECM proteins without any cellular presence. More intense staining was restricted to vessels in normal liver sections (**K**,**M**). Scale bars: 50 μm. Scanning electron microscopy (SEM) images of the normal liver (**O**) and ALS (**P**) showing the spaces previously occupied by hepatic parenchymal cells. (**O**). Scale bars: 20 μm.

Decellularization of normal livers was achieved by using a detergent-based protocol (Figure 4). The livers of control rats (Figure 4A) were perfused with water, Triton X-100, and SDS. After decellularization, a translucent, white-colored ALS was observed (Figure 4B). Furthermore, the ALS retained gross anatomical features of the native liver, making it possible to delimit each of its hepatic lobes. Macroscopically, the vascular tree was clearly visible. To investigate vascular tree preservation, the ALS was perfused through the bile duct and portal vein with phenol red and toluidine blue, respectively. After dye perfusion, we observed that the ALS showed an intact 3D vasculature (Figure 4C,D). While toluidine blue filled all the vascular segments of the portal vein that flowed into each of the hepatic lobes, phenol red reached the microvascular regions that permeated all the hepatic lobes. No dye leakage was observed. Microscopic analyses of the vasculature were also performed to confirm microvascular preservation after decellularization (Figure 4E–G). To perform microscopic vascular tree evaluation, the ALS was examined by using an intravital microscope and subjected to microscopic analysis (Figure 4E). We observed delimited blood vessels distributed normally along the hepatic lobe (Figure 4F,G). Therefore, we confirmed the preservation of the vascular tree of the scaffold at the microscopic level, even after decellularization.

Decellularization effectively removed DNA from the ALS (normal liver: 1813.0 ± 125.6 ng DNA/mg vs. ALS: 16.93 ± 4.00 ng DNA/mg; *p* < 0.0001) (Figure 4H). Compared to normal liver tissue (Figure 4I), H&E staining revealed the absence of nuclei and cytoplasmic components in ALS (Figure 4J). In addition, Sirius red (Figure 4L) and Gomori’s trichrome (Figure 4N) staining revealed ECM preservation after decellularization. More intense staining was restricted to areas around the vessels composed mainly of collagen I in the normal tissue sections (Figure 4K,M). Scanning electron microscopy analysis confirmed the elimination of nuclei, revealed collagen fiber integrity, and showed general tissue preservation of the micro- and ultra-architecture, with maintenance of the arrangement and conservation of the three-dimensional liver ECM (Figure 4P).

### 3.4. Acellular Liver Scaffolds Can Be Repopulated by Cells after Transplantation into Cirrhotic Rats

**Figure 5:**
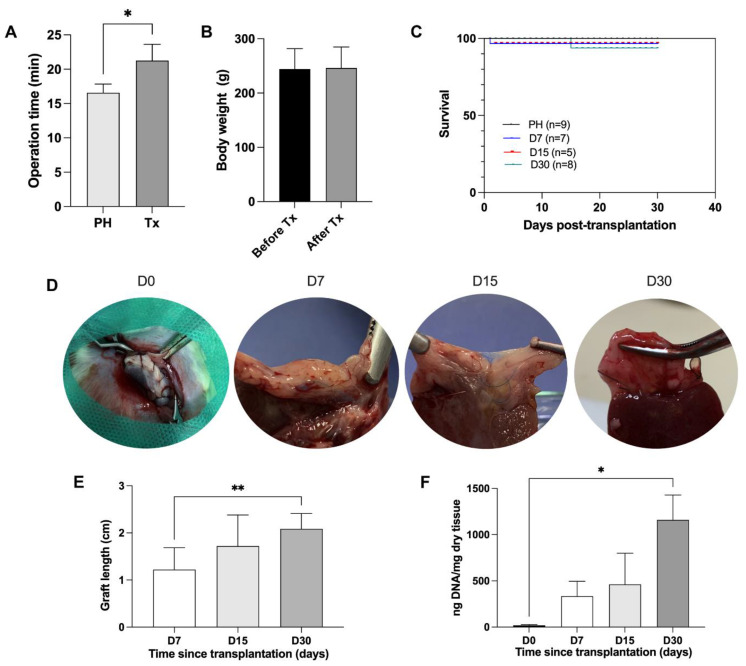
**Acellular liver scaffold transplantation into rats with cirrhosis.** Operation time (partial hepatectomy group, PH, *n* = 9 vs. transplanted group, Tx, *n* = 20) (**A**), body weight (**B**), and probability of survival of recipient animals subjected to ALS transplantation 7, 15, and 30 days post-transplantation compared to only partially hepatectomized rats (PH) (**C**) (log-rank: Mantel–Cox test). Gross appearance of ALS at transplantation (D0) and D7, D15, and D30 days post-transplantation (**D**). Graft size analysis at 7, 15, and 30 days post-transplantation (**E**) (*n* = 5). DNA content quantification in transplanted grafts before and 7, 15, and 30 post-transplantation (*n* = 3). Significant differences were assessed by using Student’s *t*-test (**A**,**B**), one-way ANOVA followed by Tukey’s test (**E**), and Friedman’s test (**F**).* *p* < 0.05, ** p < 0.005.

Cirrhotic rats were used as recipients to promote ALS partial orthotopic transplantation (Figure 5). The transplanted graft (ALS) was evaluated 7, 15, and 30 days after transplantation.

The mean operative duration was significantly higher (21.26 ± 2.38 min) in the transplanted group (Tx) than in the partially hepatectomized cirrhotic rats (PH; 16.58 ± 1.27 min) subjected to only partial hepatectomy (Figure 5A). All rats were alert and were able to perform normal activities after transplantation. Body weight measurements (before Tx 244 ± 37.91 g and 7, 15 and 30 days after Tx 246.2 ± 38.64 g) revealed that the transplantation did not affect animal feeding (Figure 5B). In addition, the transplanted rats had normal glossy hair without local signs of skin inflammation.

No significant difference was observed between the PH and Tx groups in terms of survival (Figure 5C). While no deaths were observed in the PH group, two deaths were observed in the first 24 h after transplantation and one death was observed 7 days after transplantation in the Tx group, totaling three deaths (15%, 3/20).

Following exploratory laparotomy after euthanasia, macroscopic analysis revealed the assembly of a soft textured, brownish colored, and vascularized tissue connected to the recipient rat median lobe (Figure 5D). The scaffold was still visible from the surrounding cirrhotic liver at 7, 15, and 30 days after transplantation. In addition, a significant increase in graft length was observed 7, 15 and 30 days after transplantation (Figure 5E). To investigate whether the increase in graft size could be associated with the degree of recellularization after in vivo transplantation, we performed a DNA content quantification analysis (Figure 5F). An increase in DNA content was observed after the graft was transplanted, confirming cell repopulation into the graft after in vivo transplantation in recipient cirrhotic rats. A significant increase in the amount of DNA was detected 30 days after transplantation.

### 3.5. Cirrhotic Rats Can Promote In Vivo Recellularization of Acellular Liver Scaffolds after Transplantation

**Figure 6:**
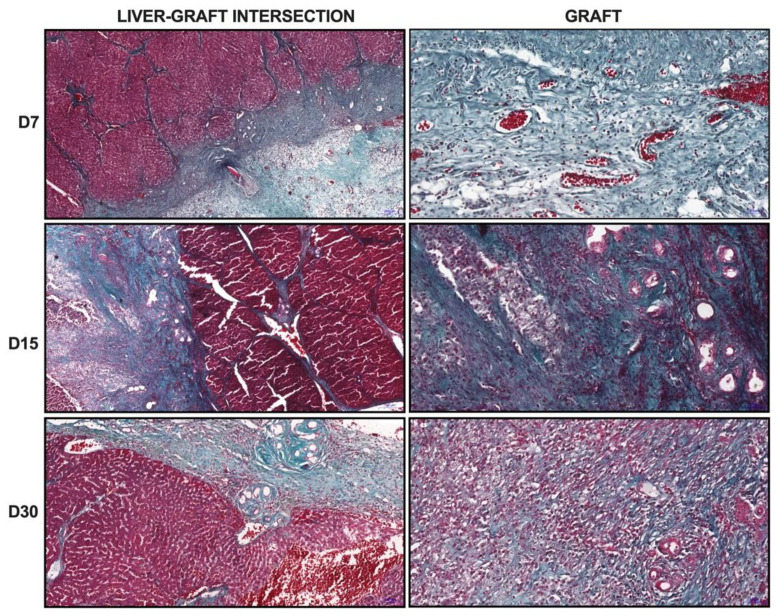
**Microscopic view of the transplanted graft tissues**. Histological sections stained with Gomori’s trichrome showed transplanted ALS in cirrhotic liver. The images showed an intersection zone that delimited the cirrhotic liver and the transplanted graft. Higher magnification images from ALS at 7, 15, and 30 days post-transplantation showed cell migration, distribution, and graft repopulation. Scale bars: intersection, 200 μm; graft, 50 μm.

Histological analyses were performed to investigate whether cirrhotic rats were able to promote in vivo recellularization of the transplanted ALS. First, we attempted to identify the transplanted graft at 7, 15, and 30 days after transplantation into recipient cirrhotic rats. Histological sections stained with Gomori’s trichrome revealed the transplanted graft at all analyzed time points (Figure 6). The red- and green-based stains were able to show and delimit two different but connected areas, namely the cirrhotic recipient liver (red) and the transplanted graft (green). In addition, an intersection area that divides these two tissues was also observed. Collagen was clearly highlighted by green staining within the transplanted graft area, whereas its distribution was restricted to the fibrous septa in the cirrhotic recipient liver. At 15 and 30 days after transplantation, more evident red staining was observed in the transplanted graft, suggesting an increase in cell migration, distribution, and repopulation.

**Figure 7:**
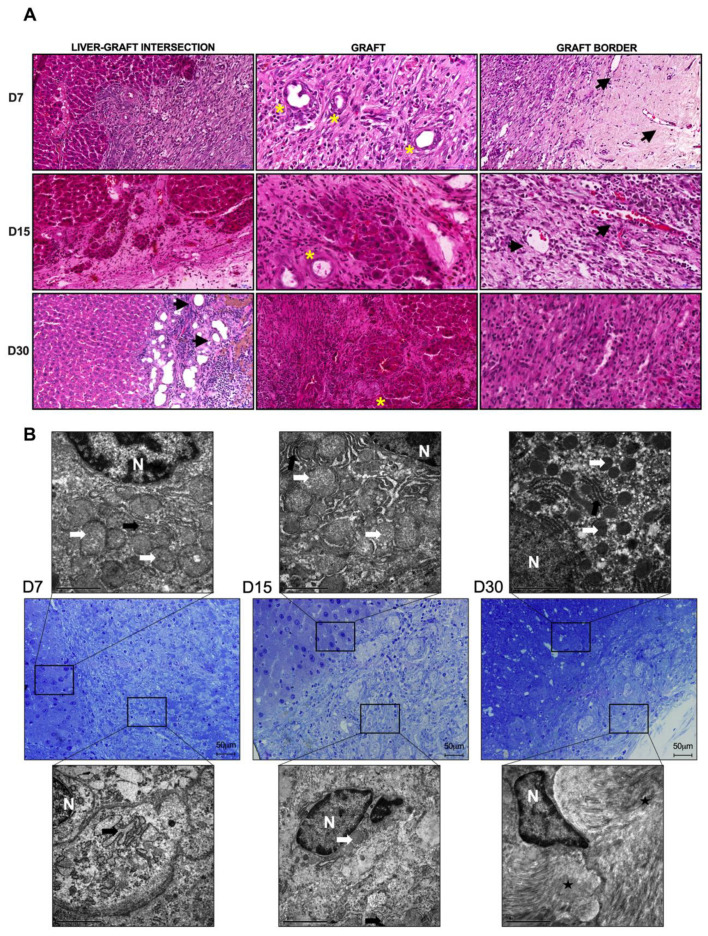
**Cirrhotic rats promote in vivo recellularization of ALS after partial orthotopic transplantation.** Microscopic view of tissue sections stained with H&E showing the intersection area, the graft, and graft border 7, 15, and 30 days post-transplantation, and confirming complete ALS recellularization by different types of cells. Blood vessel-like structures (black arrows) with red blood cells inside and bile duct-like cavities are also evident (yellow asterisks) (**A**). Scale bars: 50 μm. TEM analysis of semi-thin tissue sections (stained with toluidine blue) showed that ALS was recellularized by cells with ultrastructural similarities to those of normal liver tissue (**B**). N, nuclei; white arrows, mitochondria; black arrows, rough endoplasmic reticulum; stars, collagen fibers. Scale bars: 2 μm.

To confirm cell migration, distribution, and graft repopulation after transplantation in cirrhotic rats, H&E staining was performed. The transplanted graft was depicted in the following three different areas: (i) the intersection area between the cirrhotic liver and the transplanted graft, (ii) the graft, and (iii) the graft border (Figure 7). Cell migration into the transplanted graft was evident at early time points. Histological sections stained with H&E revealed intense graft recellularization 7, 15, and 30 days after transplantation. We observed that the transplanted graft was progressively repopulated by a large number of cells that were distributed along the graft. While there was evident cell repopulation near the intersection area between the cirrhotic liver and the graft at 7 days after transplantation, a more robust distribution of cell nuclei was observed 15 and 30 days after transplantation. At these time points, cell invasion and migration had repopulated distant regions of the intersection zone, such as the graft border (Figure 7A). These data correlate with the observed increase in DNA content 30 days after transplantation.

In addition to confirming that ALS can be recellularized after transplantation in cirrhotic rats, we also observed the presence of diverse cell nuclei in the transplanted graft, including areas where confluent cells were arranged that originated as islands, suggesting heterogeneous recellularization by different types of cells, including hepatocytes (Figure 7A). Infiltration by leukocytes and fibroblastic cells was observed at all the analyzed time points. Furthermore, blood vessel-like structures (black arrows) and bile duct-like cavities (yellow asterisks) were also evident at early time points. These structures were not restricted to the intersection area, but were also observed in the graft parenchyma and near the border. Red blood cells were detected inside the blood vessel-like structures at all analyzed time points, suggesting blood flow in the transplanted graft at 7, 15 and 30 days after transplantation. Interestingly, intense vascularization was observed in the intersection zone 30 days after transplantation (Figure 7A).

Ultrastructural examination confirmed that ALS was recellularized by several cell types and that these cells showed ultrastructural features similar to those of normal liver cells (Figure 7B, upper panel). A well-condensed cytoplasm with normal organelle distribution was observed. Hepatocyte microstructures, including rough endoplasmic reticulum and mitochondria, as well as ECM proteins and collagen fibers, were observed at 7, 15 and 30 days post-transplantation (Figure 7B, bottom panel).

**Figure 8:**
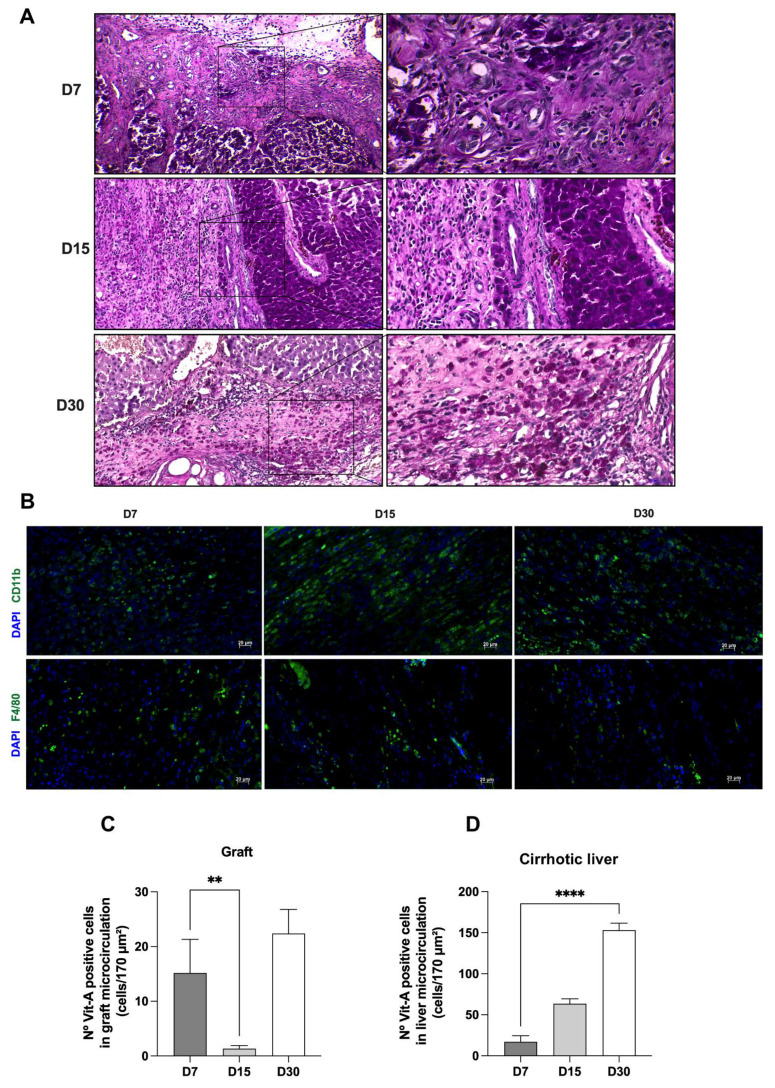
**ALS can be recellularized by parenchymal and non-parenchymal liver cells after transplantation in cirrhotic rats.** Histological sections stained with periodic acid–Schiff (PAS) stain showed glycogen-positive cells at all analyzed time points (**A**). Scale bars: 50 μm. Immunofluorescence analysis confirmed the presence of CD11b- and F4/80-positive cells in the scaffold 7, 15, and 30 days post-transplantation (**B**). Scale bars: 20 μm. Number of vitamin A-positive cells in the graft (**C**) and liver (**D**) microcirculation at 7, 15, and 30 days post-transplantation (significant differences were assessed by applying Student’s *t*-test; D7, *n* = 6; D15, *n* = 3; D30, *n* = 5, ** *p* < 0.05, **** p < 0.0001).

We also confirmed ALS recellularization by healthy morphology and positive staining for glycogen. Histological sections stained with periodic acid–Schiff (PAS) revealed that part of the transplanted graft had been repopulated by parenchymal cells that assembled hepatic-lobule-like structures (Figure 8A). PAS staining confirmed glycogen storage in cells and suggested the presence of hepatocytes at all the analyzed time points.

We performed immunofluorescence analysis to investigate non-parenchymal cell recellularization after ALS transplantation in cirrhotic rats (Figure 8B). In vivo transplantation of ALS was associated with the presence of CD11b-expressing cells at all analyzed time points. In particular, CD11b expression followed a time-dependent trend, with maximum expression at 15 days post-transplantation. F4/80-positive cells were also detected at all the analyzed time points (Figure 8B). The maximum expression was detected 7 days post-transplantation. These results confirmed leukocyte recruitment, which could include monocytes, neutrophils, macrophages and granulocytes, in addition to liver Kupffer cells. These results agree with the observations of H&E-stained tissue sections (Figure 6).

We also assessed the engraftment of hepatic stellate cells in ALS and native liver after transplantation using vitamin A storage detection by intravital microscopy. In vivo transplantation of ALS cells was associated with the presence of vitamin A-expressing cells at all analyzed time points (Figure 8C). However, vitamin A storage by stellate cells followed a time-dependent trend, with a significant decrease in vitamin A storage at 15 days post-transplantation, with recovery at 30 days post-transplantation. A higher number of vitamin A-expressing cells was found at 7 and 30 days post-transplantation. This profile revealed reversible behavior in response to ECM–cell interactions and tissue remodeling after transplantation. In parallel, we also investigated hepatic stellate cell behavior in cirrhotic recipient livers (Figure 8D). Interestingly, the number of vitamin A-positive cells significantly increased after transplantation, suggesting that ALS transplantation could induce a remodeling and regenerative effect on the cirrhotic liver that was initiated 7 days after transplantation.

### 3.6. Acellular Liver Scaffold Recellularization Is Mediated by Tissue Cell Activation, Proliferation, and Apoptosis after Transplantation in Cirrhotic Rats 

**Figure 9:**
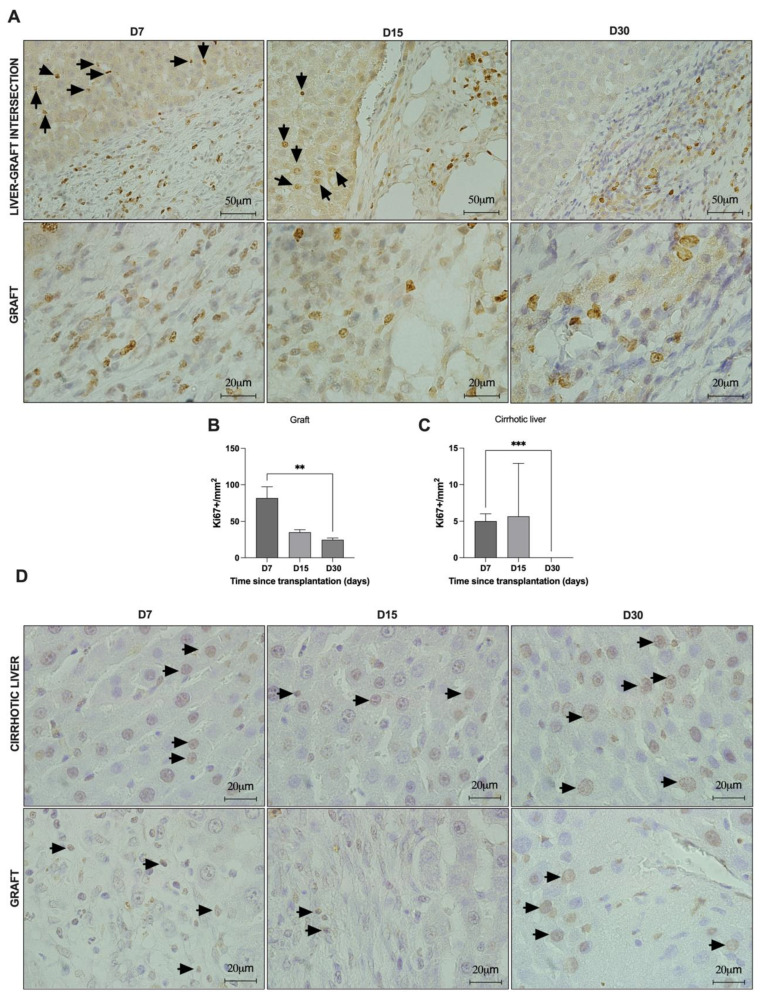
**ALS transplantation promotes tissue remodeling in both cirrhotic liver and grafts.** Immunostaining (**A**) and quantification of Ki67-positive cells in the graft (**B**) and in the intersection area (liver-graft intersection) (**C**) at 7, 15, and 30 days post-transplantation. TUNEL-positive cells in cirrhotic liver (**D**, **top images**) and graft (**D**, **bottom images**) at 7, 15, and 30 days post-transplantation. Black arrows indicate Ki67- and TUNEL- positive cells. Scale bars: 20 μm, ** *p* < 0.05, *** *p* = 0.0001.

Tissue cell activation was confirmed by Ki67 staining (Figure 9). Soon after ALS transplantation, resident liver cells located near the intersection zone began proliferating and migrating toward the ALS (black arrows) (Figure 9A). Proliferating cells were detected in recipient cirrhotic livers at 7 and 15 days after ALS transplantation. No Ki67-positive cells were detected in recipient cirrhotic liver 30 days post-transplantation (Figure 9A,C). In contrast, cell proliferation was prolonged in ALS, and Ki67-positive cells were detected at all analyzed time points, although proliferating cells decreased significantly at 30 days post-transplantation (Figure 9A,B). In addition to confirming that the transplanted graft was gradually recellularized by resident and proliferative cells, we also observed graft remodeling by using TUNEL analysis (Figure 9D). Apoptotic nuclei were detected in cirrhotic liver and graft tissues at all time points.

### 3.7. Intense Bile Duct Proliferation Contributes to ALS Recellularization after Transplantation in Cirrhotic Rats

**Figure 10:**
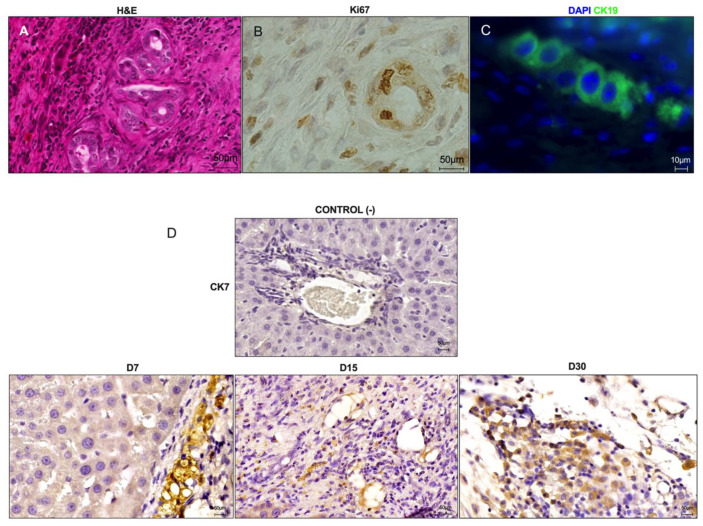
**Intense bile duct proliferation contributes to ALS recellularization after transplantation in cirrhotic rats.** Histological sections stained with H&E show intense bile duct proliferation 7 days after ALS transplantation (**A**). Ki67 immunostaining shows proliferating cells in the bile duct structures 7 days post-transplantation (**B**). CK19-positive cells were distributed in ALS 30 days post-transplantation (scale bar: 10 μm) (**C**). CK7 immunostaining revealed bile duct structures at 7 and 15 days post-transplantation. CK7-positive cells were distributed in the ALS parenchyma 30 days post-transplantation (**D**). Scale bars: 50 μm.

Histological examination revealed that ALS was recellularized by epithelial cells arranged in an aligned configuration (Figure 10). These cells assembled biliary structures distributed within the transplanted ALS at all analyzed time points (Figure 10A). Immunostaining analysis revealed that these cells were Ki67-positive (Figure 10B). Intense bile duct proliferation appeared on the histological slices and was more evident at the intersection zone between the ALS and native cirrhotic liver, both stained with H&E and marked with CK7 at 7 days post-transplantation, suggesting bile duct regeneration (Figure 10A,D). A decrease in bile duct cell proliferation and distribution was qualitatively observed on day 15 post-transplantation. Thirty days after transplantation, CK19- and CK7-positive cells were detected throughout the ALS and were not restricted to biliary structures (Figure 10C,D). 

### 3.8. Acellular Liver Scaffold Can Sustain Blood Circulation after Transplantation in Cirrhotic Rats

**Figure 11:**
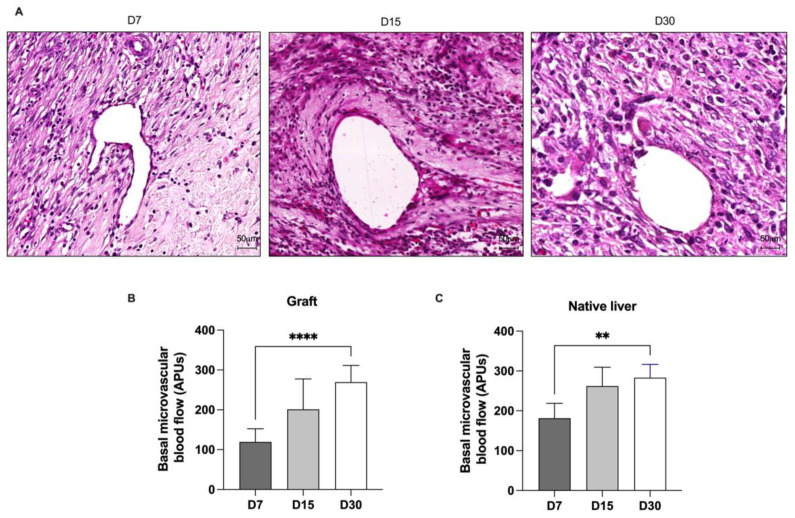
**Acellular liver scaffolds promote angiogenesis and sustain blood circulation after transplantation in cirrhotic rats**. Histological sections stained with H&E showed blood vessel structures 7, 15, and 30 days post-transplantation (**A**). Measurement of basal microvascular blood flow in the graft (**B**) and cirrhotic liver (**C**). Significant differences were assessed by using Student’s *t*-test analysis (** *p* < 0.05, **** *p* < 0.0001); D7, *n* = 6; D15, *n* = 3; D30, *n* = 5. Scale bars: 50 μm.

We observed numerous blood vessels within the entire transplanted graft, including the surrounding intersection zone and the graft border (Figure 7 and Figure 11). Histological analysis of the ALS suggested widespread angiogenesis at all the analyzed time points (Figure 11A). Angiogenesis was clearly observed throughout the ALS; in particular, many small vessels, including arterioles and venules, were observed in the transplanted ALS. In addition, all vessels were limited by aligned cells, suggesting the presence of endothelial cells. Encouraged by these observations, we investigated whether these vessels were able to sustain blood circulation, by using microcirculation analysis. Basal microvascular blood flow was detected in the transplanted graft at all the analyzed time points (Figure 11B). Microvascular blood flow continued to increase over time, and a significant increase in this parameter in graft tissue was observed 30 days post-transplantation. In addition to graft microvascular blood flow analysis, we also investigated whether microvascular blood flow in cirrhotic liver improves after ALS transplantation. Indeed, we found that basal microvascular blood flow in cirrhotic livers also increased after ALS transplantation (Figure 11C).

### 3.9. Acellular Liver Scaffold Recellularization Contributes to Extracellular Matrix Production and Remodeling after Transplantation in Cirrhotic Rats

**Figure 12:**
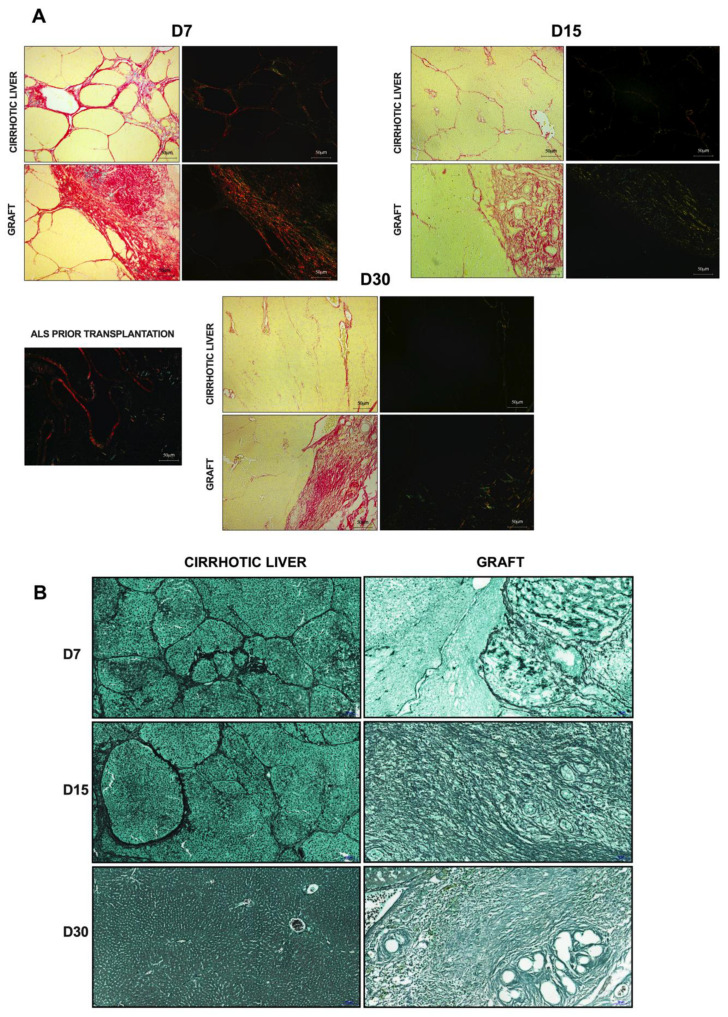
**Acellular liver scaffold recellularization contributes to ECM production and remodeling after transplantation in cirrhotic rats.** Sirius red staining under bright (left) and polarized light (right) shows collagen I and III distribution in ALS prior to transplantation, in the graft and cirrhotic liver at 7, 15, and 30 days post-transplantation (**A**). Reticulin staining revealed reticular fibers in the graft and cirrhotic liver at 7, 15, and 30 days post-transplantation (**B**). Scale bars: 50 μm.

The production of ECM proteins is essential for the assembly of new liver tissue after transplantation. We performed Sirius red staining analysis to investigate ECM deposition after ALS transplantation (Figure 12). Tissue sections derived from ALS and stained with Sirius red showed a red-stained ECM composed mainly of fibrillar collagens (predominantly types I and III) (Figure 4L). Under polarized light, these compounds were observed in orange and green, respectively (Figure 12A). Histological examination at 7 days post-transplantation revealed that the transplanted graft was composed predominantly of collagen I. In parallel, we also observed that the cirrhotic liver stained strongly with Sirius red, indicating a condensed ECM originating from a nodular configuration. On the other hand, histological examination revealed that the ECM protein composition changed 15 and 30 days post-transplantation (Figure 12A). More remarkably, green-based staining under polarized light was observed in the transplanted graft at these time points, suggesting ECM remodeling after transplantation in cirrhotic rats. These alterations were also observed in cirrhotic livers. A decrease in ECM deposition was observed 15 and 30 days after ALS transplantation. At these time points, Sirius red staining was more abundant around the vessels, and polarized light revealed that the ECM was predominantly composed of collagen III. Histological analysis (H&E and Sirius red) staining of livers from partial hepatectomized (PH) rats revealed extensive fibrosis scars and immune cell infiltration at all analyzed time points (see Figure S1).

In addition, we performed reticulin staining to investigate reticular fiber disposition in the ALS and the cirrhotic livers after transplantation (Figure 12B). Histological evaluation revealed that the cirrhotic liver was also marked by reticular fibers (in black) that were condensed to form denser septa, where hepatocytes were arranged in regenerative nodules 7 days after ALS transplantation. Reticular fibers were also detected 15 days post-transplantation; however, the throughput of the liver parenchyma was reduced at this time point. In contrast, very thin reticular fibers were detected in the cirrhotic livers 30 days post-transplantation. In parallel, we also detected reticular fibers in the transplanted grafts 7, 15, and 30 days post-transplantation. At all the analyzed time points, very thin and delicate strands of collagen III that assembled into well-distributed three-dimensional networks were observed (Figure 12B). In addition, we found that the number of reticular fibers increased after transplantation, suggesting that cell engraftment also contributes to ECM production and remodeling.

### 3.10. Acellular Liver Scaffold Transplantation Attenuates Liver Cirrhosis

**Figure 13:**
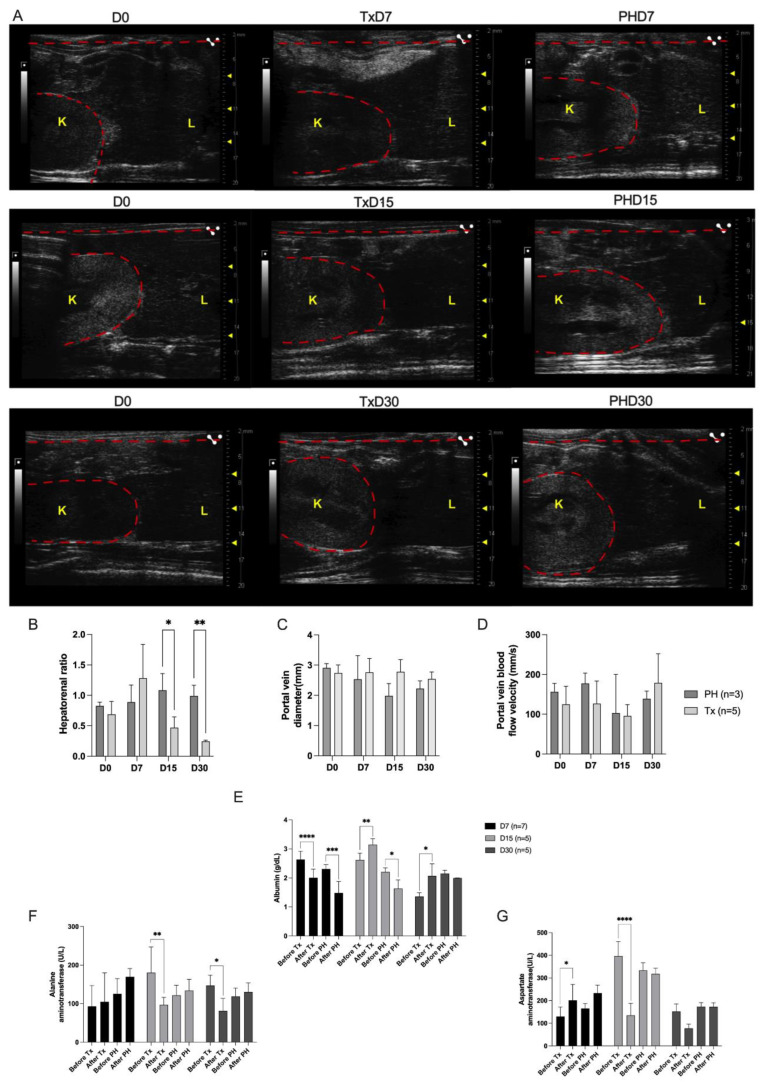
**Acellular liver scaffold transplantation attenuates liver cirrhosis and contributes to the functional recovery of cirrhotic livers.** Ultrasonographic images were derived from transplanted cirrhotic (Tx) and partial hepatectomy (PH) animals 7, 15, and 30 days post-transplantation (**A**). Measurements of the hepatorenal ratio (**B**), portal vein diameter (**C**), and portal vein blood flow velocity (**D**). L: Liver. K: Kidney (both are delimited by dashed red lines). Albumin (**E**), alanine (**F**), and aspartate aminotransferase (**G**) serum biochemical analyses in cirrhotic animals before and 7, 15, and 30 days post-transplantation (Tx) or partially hepatectomized (PH) animals. Significant differences were obtained after two-way ANOVA followed by Šídák’s multiple comparison test analysis (* *p* < 0.05, ** *p* < 0.005, *** *p* < 0.001, **** *p* < 0.0001).

Partial hepatectomized cirrhotic rats (PH) and transplanted cirrhotic rats (Tx) were subjected to non-invasive ultrasound analysis before (D0) and 7, 15 and 30 days post-transplantation (Figure 13). Collagen accumulation increased parenchymal echogenicity and contributed to the irregular liver surfaces in cirrhotic recipient rats. As a result, the livers appeared to be altered in relation to the renal cortex, becoming hyperechogenic (D0). For this reason, a comparison between the echogenicity patterns of the hepatic (indicated as “L” in Figure 13) and renal parenchyma (indicated as “K” in Figure 13) was evaluated. After 7 days post ALS transplantation, the livers were isoechogenic in relation to the renal parenchyma, and liver echogenicity did not decrease and was similar to that detected in the PH D7 group. On the other hand, ultrasound images revealed that the liver echogenicity decreased at 15 and 30 days, becoming hypoechogenic in relation to the renal parenchyma after ALS transplantation, when compared to the PH group at the same time points. In agreement with the ultrasound images, the hepatorenal ratio was significantly lower in the Tx groups at 15 and 30 days post transplantation than in the PH group at the same time points (Figure 13B). No significant differences were observed in the portal vein diameter (Figure 13C) and portal vein blood velocity (Figure 13D) at 7, 15 and 30 days post-transplantation.

### 3.11. Acellular Liver Scaffold Transplantation Contributes to Functional Cirrhotic Liver Recovery

To investigate whether ALS transplantation could affect liver function and injury parameters, we assessed recipient serum biochemical parameters (Figure 13). Biochemical analysis revealed that ALS could restore albumin levels at 15- and 30-days post-transplantation (Figure 13D). A significant decrease in albumin levels was observed 7 days post-transplantation; however, these levels recovered and significantly increased 15 and 30 days post-transplantation. In contrast, a significant decrease in albumin levels was observed in the partially hepatectomized rats at 7 and 15 days. The ALS also contributes to decreased ALT and AST levels after transplantation. Seven days after transplantation, no significant difference in ALT serum levels was observed before or after ALS transplantation (Figure 13E). In contrast, a significant decrease was observed in ALT serum levels at 15 and 30 days post-transplantation. In parallel, biochemical analysis revealed a significant increase in AST serum levels 7 days post-transplantation, followed by a significant decrease in AST levels 15 days post-transplantation (Figure 13F). Thirty days after transplantation, no significant difference was observed in serum AST levels before and after ALS transplantation (Figure 13G).

## 4. Discussion

In this study, we focused on applying acellular liver scaffolds obtained through decellularization to investigate ECM-based acellular and non-immunogenic material as a template for the in vivo recellularization of healthy cells after transplantation into cirrhotic rats. Here, we investigated the following open questions: (a) whether the body can recellularize the ALS after in vivo transplantation; (b) whether healthy cells can be recruited; (c) whether the transplanted scaffold can be affected by the cirrhotic liver that received it, either becoming cirrhotic or remaining a healthy template for healthy cell growth; and (d) whether the cirrhotic liver changed after ALS transplantation.

To address these questions, our first set of experiments focused on an efficient method for inducing liver cirrhosis in rats. Using a well-established rodent model of liver cirrhosis based on CCl_4_ administration in association with ethanol, we found that cirrhotic animals developed an inflammatory process that increased hepatic stellate cell activation and promoted the deposition of fibrous ECM compounds. This increase in ECM deposition and consequent fibrous scar formation leads to vascular alterations in cirrhotic livers, resulting in decreased blood flow flux and an increased portal vein diameter, liver echogenicity, and hepatorenal ratio. In addition to the histological and ultrasonographic alterations, our cirrhosis model also affected the biochemical parameters, resembling human cirrhosis. Subsequently, we produced the ALS to perform transplantation in cirrhotic rats.

Decellularization is an efficient method for generating cell-free tissues [15]. In this study, we applied continuous perfusion of a chemical detergent to eliminate the cellular components of normal rat livers. Our protocol provided acellular liver tissue with a preserved three-dimensional native liver shape, vascular tree, and ECM components from the native liver. Our ALS was employed in a strategy for in vivo organ engineering using partial orthotopic liver transplantation [16] to investigate and harness the innate capability of the recipient body to promote cell recruitment and full recellularization, resembling new liver tissue. These aspects were similarly explored by Naeem and colleagues [7] in a study where the ALS produced by sodium lauryl ether sulfate perfusion was transplanted into Sprague Dawley rats, and by Shimoda and colleagues [10] to investigate the contribution of transplanted ALSs in regeneration after hepatectomy in mini pigs. In our study, to advance closer to clinical application, we addressed these questions using a liver cirrhosis model. To our knowledge, this is the first study to address the endogenous recruitment of liver cells into cirrhotic recipient rats with transplanted ALSs. Here, we observed that the ALS was recellularized 7, 15, and 30 days post-transplantation. This recellularization was time-dependent and cell migration and recruitment occurred gradually. While cell engraftment was more restricted to the intersection area at 7 days, a greater number of cells was present in the scaffold at 15 days and they were distributed more widely throughout the scaffold, achieving complete recellularization and repopulation of the scaffold edges within 30 days post-transplantation. This rapid recellularization capability could benefit patients who undergo hepatectomy.

The great potential of acellular scaffolds derived from decellularized organs was first demonstrated by Taylor and colleagues in 2018. In vivo organ engineering was achieved by heterotopic transplantation of acellular heart scaffolds into pigs and bovine recipients. They observed that acellular heart scaffold transplantation promoted endothelial cell adhesion to the vessel lumen, in addition to cardiomyocyte engraftment and tissue formation within the graft. In this regard, they harnessed the body’s own recuperative powers to achieve recellularization, one of the greatest hurdles in tissue engineering [17].

In our study, the transplanted scaffolds were completely recellularized by different cell types. We observed intense leukocyte engraftment, especially at the early time points. We first asked whether this could have been stimulated by the G-CSF treatment that preceded ALS transplantation; however, this cell mobilization was also observed when the transplant was performed in recipient animals that did not receive G-CSF [7,10]. These observations led us to anticipate that a granulocyte-specific component may orchestrate ALS recellularization. Our findings agree with those previously reported by Naeem in healthy rats [7] and Shimoda in healthy mini pigs [10]. Therefore, a granulocyte-specific component also orchestrated the ALS recellularization in healthy and cirrhotic recipients. In addition to granulocyte-component-mediated recellularization in healthy and liver disease contexts, leukocyte engraftment was also observed in other tissues, such as when an acellular diaphragmatic scaffold was produced and transplanted into recipient C57BL/6 mice [3] and when an acellular uterus scaffold was transplanted into Sprague Dawley recipient rats [4]. Although acellular scaffolds are considered to be immune-privileged, it is widely known that acellular scaffold transplantation is characterized by immediate mononuclear cell recruitment and engraftment [18,19]. However, cumulative evidence has shown that the presence of growth factors and cytokines within the acellular scaffold is responsible for the rapid shift of this immune response into a pro-regenerative response where inflammatory cells, including monocytes and liver-resident macrophages, restore internal communication with fibroblasts, epithelial, endothelial, and stem or tissue progenitor cells [20,21,22,23]. Taken together, this pro-regenerative response is amplified by cellular stimulation and recruitment [3,24]. It is also worth noting that immune response activation is one of the most important key markers for promoting tissue regeneration [25,26].

Despite leukocyte engraftment, other cell types such as fibroblasts, Kupffer cells, and hepatocytes migrated within the graft, showing that the ALS was a good niche for cell repopulation and proliferation of different types of cells at all the analyzed time points. Although engraftment of hepatocytes has been observed, extended follow-up analysis must be performed to determine if the number of these cells increases after transplantation. In addition, we observed the engraftment of hepatic stellate cells. In our study, stellate cells were useful for demonstrating the tissue remodeling that ALS undergoes after transplantation. Interestingly, the number of activated stellate cells was increased at 15 days post-transplantation, suggesting a pro-regenerative phase. In parallel, the number of activated stellate cells was decreased at 30 days post-transplantation, confirming the anti-inflammatory effect of transplanted ALS.

In addition to detecting the presence of these cells, we also observed cholangiocytes in ALS. Similarly, Naeem and Shimoda observed the engraftment of cholangiocytes in transplanted ALS. These cells were organized into duct structures that assembled the bile ducts into the transplanted grafts. Importantly, we observed intense bile duct proliferation in the grafts after transplantation. This finding indicates that a bile duct activation pathway can compensate for ALS recellularization after transplantation in cirrhotic rats. The biliary system is known to be involved in liver regeneration and repair, especially after partial hepatectomy [27,28]. Likewise, biliary-derived cells can expand and differentiate into hepatocytes. This intense bile duct proliferation might also contribute to ALS recellularization after transplantation through a reparative complex pathway called the ductular reaction [29,30,31]. This supportive evidence agreed with our histological and immunohistological findings, which showed that bile duct proliferation was more intense 7 days post-transplantation, but decreased at 15 and 30 days. Especially at 30 days, we found that many CK7- and CK19-positive cells were distributed throughout the parenchyma of the ALS and no longer in ductular structures. Therefore, our data suggest a correlation of the ductular reaction with a positive outcome of ALS transplantation in recipient cirrhotic rats in early time points after transplantation. Our results showed that this event was important to drive recellularization by liver parenchyma cells, which may have arisen from these proliferating structures. Furthermore, cumulative evidence has shown that CK19 is also a marker of stem/progenitor cells, suggesting the presence of stem and progenitor cells in ALS after transplantation [27,28,29,30,31].

In vivo implantation of the ALS gave rise to changes in the characteristics of the recipient cirrhotic livers. These changes resulted from orchestrated events and processes involved in tissue remodeling, angiogenesis, and the immune response promoted by liver injury. To date, little is known about the origin of the cells after acellular scaffold transplantation. Our findings demonstrated proliferative cells in cirrhotic recipient livers 7 and 15 days after ALS transplantation, suggesting that resident hepatocytes proliferate to ensure ALS recellularization at early time points after transplantation. In addition to proliferative events, we also observed that apoptotic events were present in the transplanted graft and in the cirrhotic recipient livers, suggesting that both cell proliferation and cell death were involved in tissue connection and remodeling. These observations highlight that, in addition to the immune response and bile duct proliferative events, the ALS was also recellularized by resident cells that arose from proliferative hepatocytes in the cirrhotic livers. The pro-regenerative response activated by ALS transplantation also contributes to the attenuation of liver cirrhosis. For example, the ultrasonographic parameters improved, in agreement with the histological observations. Moreover, the biochemical parameters also indicated recovery in liver function and attenuation of liver injury markers. In contrast, cirrhotic animals subjected to partial hepatectomy were not able to restore their biochemical and ultrasonographic parameters.

Our findings showed that at all the analyzed time points, the transplanted ALS ensured a pro-regenerative microenvironment, attracted vessels from the host cirrhotic tissue, and promoted stem and progenitor cell recruitment to the ALS after transplantation. These events were beneficial not only for the ALS, but also for promoting tissue remodeling in cirrhotic livers. We observed well-distributed blood vessel-like structures in the transplanted ALS at all the analyzed time points. In some tissue sections, we also observed red blood cells in the vessels, suggesting the presence of blood flux. In agreement with our histological findings, we observed that the transplanted ALS was able to support blood flow at all analyzed time points. The angiogenic potential was improved at the time of transplantation, reaching a higher level of blood flow circulation at 30 days post-transplantation. These angiogenic events effectively facilitated the migration and repopulation of resident cirrhotic liver cells. Certainly, blood flow microcirculation was important to promote nutrient and oxygen supply to the transplanted ALS, allowing the formation of new liver tissue. Interestingly, the improvement of blood flow microcirculation in the recipient cirrhotic livers was also observed after ALS transplantation. To our knowledge, this is the first study to confirm blood flow circulation into the ALS after transplantation in recipient cirrhotic rats.

To resemble functional liver tissue, the transplanted ALS should undergo remodeling to ensure its tissue mechanical properties; therefore, the production of structural ECM proteins is crucial. The liver contains important ECM constituents, such as fibrillar collagens (types I and III) [6,14]. In this study, we observed that cells in the graft produced neo-ECMs (collagen I and reticular fibers) after ALS transplantation. This finding is consistent with those reported by Naeem and colleagues [7]. Hepatic stellate cells, fibroblasts, and immune cells, such as macrophages and neutrophils are the major cells involved in neo-ECM deposition after ALS transplantation. We also confirmed that the transplanted ALS had the capability of in vivo remodeling, regeneration, and growth. These findings were also observed in the recipient cirrhotic livers after ALS transplantation. More importantly, we confirmed that the transplanted ALS was not affected by the cirrhotic livers and remained a template for healthy cell growth. Some studies have already shown that healthy scaffolds provide a template for the growth and development of healthy cells. Ogiso and colleagues showed that healthy ALSs can sustain fetal hepatocytes and cholangiocytes after in vitro recellularization [32]. Acun and colleagues observed that healthy ALSs also enhanced the differentiation of induced pluripotent stem cells into hepatocyte-like cells and significantly increased the expression of mature hepatocyte markers [33]. On the other hand, early studies indicated that acellular cirrhotic scaffolds derived from cirrhotic rats accelerated the epithelial–mesenchymal transition, phenotype, proliferation, and drug resistance of hepatocarcinoma cells in vitro [34]. In addition, human cirrhotic liver acellular scaffolds also promoted the Smad-dependent TGF-β1 epithelial–mesenchymal transition when HepG2 cells were recellularized in vitro [35]. Thus, we did not know a priori whether cirrhotic cells derived from cirrhotic livers could repopulate ALS and assemble healthy liver tissue. Histological, biochemical, and ultrasonographic analyses confirmed that the ALS was recellularized by healthy cells to generate tissue resembling healthy liver tissue. Our findings demonstrate that the ALS obtained by decellularization is a potent scaffold for cell proliferation and differentiation induction that is capable of providing an appropriate environment for liver tissue assembly and formation.

In light of the encouraging results obtained with rats in this study, we speculate that ALSs can be readily extended to large animal models of liver disease so that in the near future, this alternative will reach patients. Our data provide proof of concept that ALSs can be transplanted into patients with liver disease, as an alternative to the rapid replacement of liver mass and as metabolic support until the patient’s own liver recovers.

## 5. Conclusions

In conclusion, our findings show that ALSs can be recellularized, even when transplanted into cirrhotic recipient rats. Furthermore, ALS transplantation enables in vivo regeneration by promoting endogenous cell recruitment, proliferation, and stimulation. The transplanted ALS was able to orchestrate immune responses, tissue remodeling, and angiogenesis events. We also conclude that the transplanted ALS was not affected by the cirrhotic livers and remained a template for healthy cell growth. In a translational context, this study demonstrates that ALSs derived from decellularization can be applied as a bioartificial liver serving as auxiliary support or transplanted into patients with liver disease undergoing hepatectomy procedures. In addition, we provide evidence that tissue engineering products can be combined with therapeutic cytokines and may offer a novel therapeutic approach for the treatment of acute and chronic liver diseases in humans.

## Data Availability

The data presented in this study are available within the article and will be provided by the corresponding author under reasonable request.

## Supplementary Materials

The following are available online at https://www.mdpi.com/article/10.3390/cells12070976/s1, Figure S1.
