# Supplementary figures and images for: Cirrhotic Liver Sustains In Situ Regeneration of Acellular Liver Scaffolds after Transplantation into G-CSF-Treated Animals
Source: Cells. 2023 Mar 23;12(7):976. doi: 10.3390/cells12070976 (PMC10093225; doi:10.3390/cells12070976)

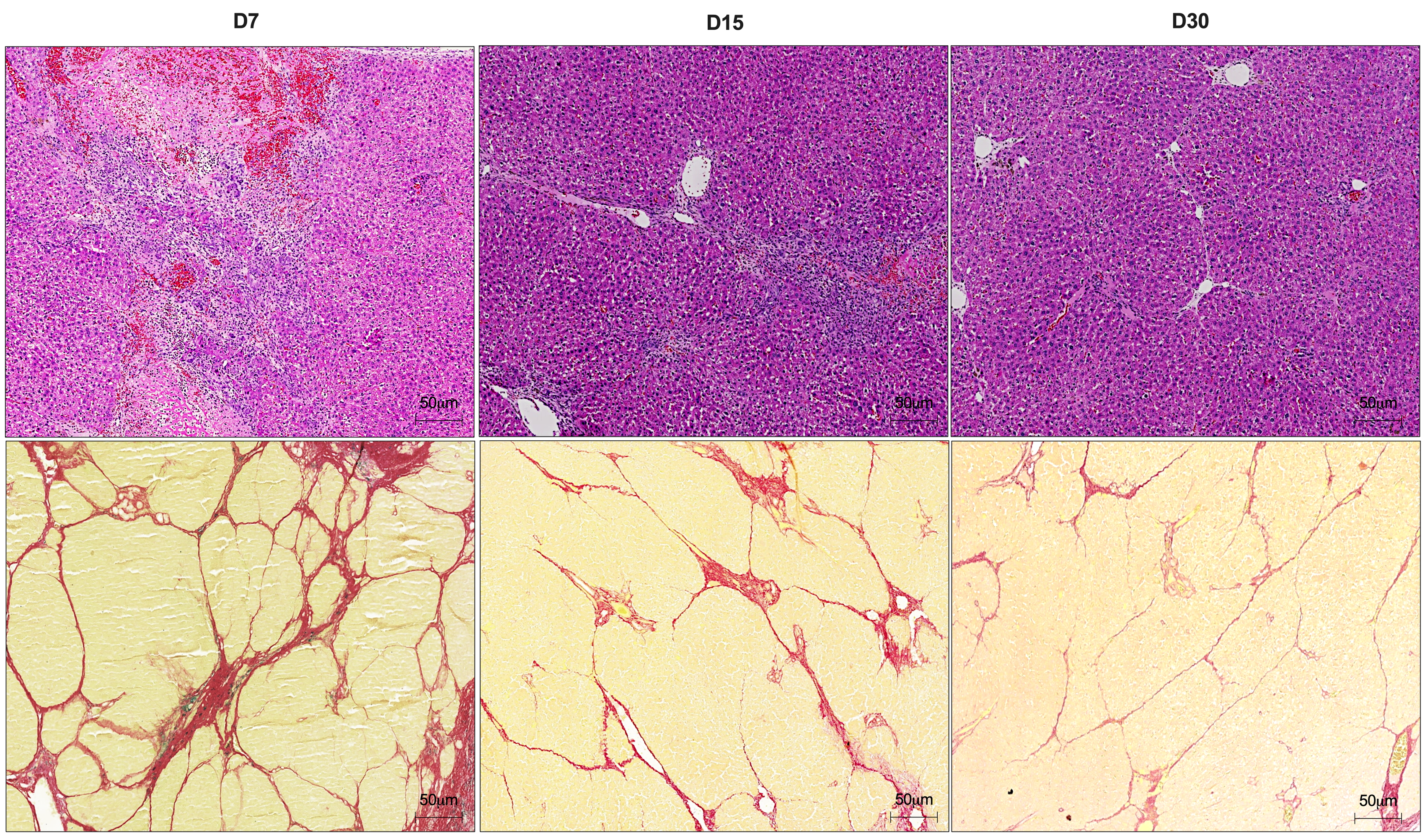

Supplement: Supplementary file 1 [file cells-12-00976-s001.zip › cells-2209858-supplementary.tiff]
